# Host Adaptation Is Contingent upon the Infection Route Taken by Pathogens

**DOI:** 10.1371/journal.ppat.1003601

**Published:** 2013-09-26

**Authors:** Nelson E. Martins, Vitor G. Faria, Luis Teixeira, Sara Magalhães, Élio Sucena

**Affiliations:** 1 Instituto Gulbenkian de Ciência, Oeiras, Portugal; 2 Centro de Biologia Ambiental, Faculdade de Ciências da Universidade de Lisboa, Lisboa, Portugal; 3 Universidade de Lisboa, Faculdade de Ciências, Departamento de Biologia Animal, Lisboa, Portugal; Stanford University, United States of America

## Abstract

Evolution of pathogen virulence is affected by the route of infection. Also, alternate infection routes trigger different physiological responses on hosts, impinging on host adaptation and on its interaction with pathogens. Yet, how route of infection may shape adaptation to pathogens has not received much attention at the experimental level. We addressed this question through the experimental evolution of an outbred *Drosophila melanogaster* population infected by two different routes (oral and systemic) with *Pseudomonas entomophila*. The two selection regimes led to markedly different evolutionary trajectories. Adaptation to infection through one route did not protect from infection through the alternate route, indicating distinct genetic bases. Finally, relatively to the control population, evolved flies were not more resistant to bacteria other than *Pseudomonas* and showed higher susceptibility to viral infections. These specificities and trade-offs may contribute to the maintenance of genetic variation for resistance in natural populations. Our data shows that the infection route affects host adaptation and thus, must be considered in studies of host-pathogen interaction.

## Author Summary

Pathogens enter their hosts through several routes, the most common being ingestion (oral infection) and breaches in the cuticle (systemic infection). Several studies have shown that these infection routes strongly affect the evolution of pathogen virulence, though little attention has been given to the role of host evolution in this process. Here, we study the effect of infection route on the evolution of host defenses, using *Drosophila melanogaster* and its natural pathogen *Pseudomonas entomophila*. Profiting from the power of experimental evolution, in which the evolution of populations is followed in real time, we show that survival of *D. melanogaster* to an oral infection increases within the first 3 generations of selection, whereas the response to systemic infection is slower. Furthermore, we show that the evolved response is specific to the route of infection and to pathogen. Indeed, flies that resist bacteria through ingestion are not protected from systemic infection with the same bacteria species, and vice versa. Also, evolution of resistance to one pathogen does not extend to infections with bacteria of different genera via the same infection route. This degree of specificity calls for more attention onto pathogen infection routes in studies of host-parasite interactions.

## Introduction

The transmission route taken by pathogens to infect their hosts has a profound impact on the evolution of host-pathogen interactions. A body of theory [1], [2], [3] and several experiments [4], [5], [6], [7] have addressed the effect of vertical or horizontal transmission on the evolution of pathogen virulence. Moreover, virulence in vector-borne or directly transmitted pathogens is expected to be differentially-affected by several factors, such as the timing of infection or inoculum size [8], [9], [10]. Recently, a meta-analysis has also shown that systemically-infecting pathogens are more virulent than those that infect via ingestion [11]. However rich this body of literature may be, it concerns the effect of transmission routes on the evolution of pathogens, not hosts (even though this implies measuring host traits, as pathogen virulence is defined as the harm imposed on hosts) [12], [13]. Pathogens that infect hosts via different routes (e.g., orally *vs* systemically) also trigger different physiological responses in hosts. This in turn may affect the evolution of host responses to pathogens, which will affect the outcome of the host-pathogen interaction. Therefore, addressing the evolutionary consequences of transmission route for host-parasite interactions calls for a characterization of its effects in the evolution of both pathogen and host.

It has been suggested that the immune response follows a hierarchical structure, starting with behavioural avoidance, through physical barriers and culminating in a humoral/cellular response [14], [15], [16]. Different infection routes will impact this cascade of events at different levels. Thus, the route taken by the pathogen will be crucial in defining the evolutionary consequences of infection to the individual and population. Yet, the distribution of variants across different levels in this cascade of events is unknown: which level is more likely to evolve in a population exposed to a particular immune challenge? If host adaptation occurs through changes in a shared downstream portion of the cascade such as the humoral effectors, then adapted populations are expected to show a positive correlated response to challenges acting on any part of the cascade. Conversely, if there is at least partial independence in the defence pathways activated by each infection route, then adaptation to pathogens infecting through different routes should be uncorrelated. Thus, testing host evolutionary responses to infection through different routes is crucial to ecological immunology as it will, (a) establish whether responses are general or specific for distinct routes of pathogen access and, (b) provide insight into which part of the defense cascade may be modified by evolution.

In recent years much attention has been given to the mechanistic distinction between resistance (capacity to limit pathogen loads) and tolerance (capacity to survive damage caused by a given pathogen load) [17], [18], [19]. Yet, although a few recent studies have determined if resistance or tolerance mechanisms are involved in insect host responses to pathogens [20], [21], [22], whether and how different transmission routes affect the evolution of these mechanisms is still unknown. Indeed, no study has yet addressed the consequences of different infection routes of horizontally-transmitted pathogens for the evolution of host responses.

Routes of infection observed in nature are paralleled by the infection protocols used in the *Drosophila melanogaster* laboratory model of insect immunity [23], [24], [25]. Traditionally, the study of *Drosophila* immunity is done with systemic infections [26], [27], [28], [29], but more recently, several studies have addressed the immune response to ingested bacteria [30], [31], [32], [33], [34], as the ecological relevance of this route of infection is most likely higher (for a review see [35]). These studies have shown that several responses are specific to the infection route, even if some overlap can be observed [30], [33], [36]. Indeed, to infect hosts, ingested pathogens need to avoid evacuation, resist oxidative burst and/or breach the epithelial gut barrier [32], [37], [38], [39]. For example, Kuraishi and co-workers [40] have found that loss of Drosocrystallin, a protein involved in the formation of the peritrophic matrix, leads to increased mortality after ingestion *of P. entomophila* and *S. marcescens*, but does not seem to play a role in systemic infections. Conversely, systemic infections bypass those defence levels [25] leading, in most cases, to virulence at much lower doses [31] and inducing melanisation responses that are not observed in oral infections [41]. However, besides the local specific response, oral infection may induce, a systemic response [31], [34], [38] although not always [30].

Because it is a model system for both invertebrate immunity [23], [42] and experimental evolution [43], *Drosophila melanogaster* stands out as the ideal organism to address the evolutionary consequences for hosts of different infection routes. In particular, recent years have witnessed the use of experimental evolution in *Drosophila* to unravel the evolution of host responses to pathogens [44], [45], [46], [47], [48]. However, all these studies concern host evolution to one specific immune challenge, and hence they do not address how different infection routes affect the host response. In the work here presented, we bridge this gap using experimental evolution on an outbred population of *D. melanogaster* responding to two routes of infection of the bacteria *Pseudomonas entomophila*. In brief, we will, (a) compare the rate of adaptation to each challenge, (b) test whether pathogen loads after infection changes with the evolutionary history of populations, (c) address whether adaptation is specific to each infection route and (d) test the generality of the response towards other pathogens.

## Results

### 1. Adaptation to *P. entomophila* oral and systemic infections

**Figure 1:**
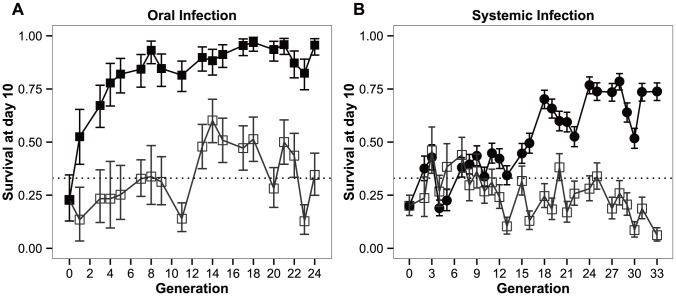
Response to selection. Experimental evolution trajectories of populations evolving with a *Pseudomonas entomophila* oral (a) or systemic (b) infection and their respective control populations. Shown is the survival of flies from each selection regime when infected with *P. entomophila* either by (a) ingestion (orally) or, (b) pricking (systemic). Closed symbols: populations evolving in presence of the pathogen; open symbols: control lines. Vertical bars correspond to standard error across means of replicate lines; the straight dotted line corresponds to the original mortality rate imposed on the populations (66%).

In figure 1, we present the survival along of the selected and control populations across 24 and 34 generations of experimental evolution, upon exposure to the natural pathogen *P. entomophila*, by oral (Figure 1a) and systemic infection (Figure 1b).

Both the selection regime and selection regime by generation effects were significant (*P*<0.0001), either in the BactOral scenario (χ^2^_1_ = 35.452 and χ^2^_17_ = 60.522 for the selection regime and selection regime by generation effects, respectively) and the BactSys scenario (χ^2^_1_ = 16.336 and χ^2^_25_ = 265.756, respectively).

Upon oral infection, the mean number of live individuals at day 10 after infection rose from the control 33% to a stable 90% after approximately 5 generations (Figure 1a). This rise is quite spectacular in that in only 3 generations the number of alive orally-infected flies had doubled (Figure 1a). Concomitantly, pairwise comparisons at each generation reveal significant differences among selection regimes for this treatment starting at generation 3 (|z|>3.072; *P*<0.05 for all comparisons beyond that generation). In contrast, selection via systemic infection with the same bacterium, only led to significant differences at generation 13 (|z|>4.160; *P*<0.001). This difference was consistently significant after generation 16 (|z|>3.887; *P*<0.01), except for generation 20 (z = 3.065; *P* = 0.05), The lines selected in presence of the pathogen never exceeded 80% survival (Figure 1b).

### 2. Pathogen loads of control and selected flies

**Figure 2:**
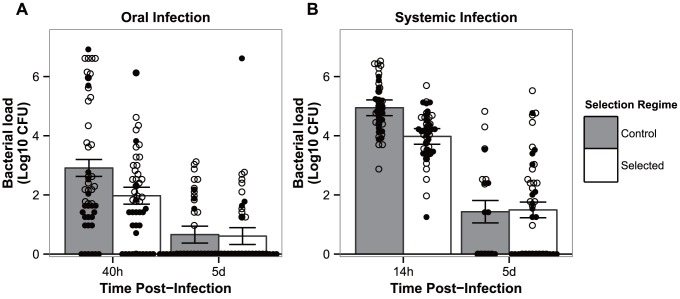
Flies have evolved resistance against *P. entomophila* infection. Bacterial loads in flies from both control populations (grey bars) and populations evolving in presence of a pathogen (white bars) when exposed to oral (a) or systemic (b) infection. Males (full diamonds) and females (empty diamonds) are represented separately. Vertical bars correspond to the standard error of the mean pathogen load of each selection regime at each time point. (N = 48, except for panel b) systemic infection on control lines after 5 days where N = 22).

Next, we asked whether the increased levels of survival observed after 24 generations of selection corresponded to differences in pathogen loads after infection. For both modes of infection and for the early time point corresponding to the onset of mortality (left bars on Figure 2a and 2b), the profile was the same, displaying a significantly higher number of bacteria in controls relatively to the evolved populations (|z| = 3.287 and 3.430, for oral and systemic infections, respectively, *P*<0.01 for both comparisons). At the later time point, after which no more death is observed between populations (right bars on Figure 2a and 2b), there were no statistical differences between bacteria titers in the two time points for each of the infection routes (|z|>0.175 for oral and systemic infections, respectively; *P* = 0.998 for both comparisons). The absolute number of bacteria was significantly reduced between the first and second time points in all treatments and selection regimes (|z|>4.883, *P*<0.001 for all pairwise comparisons) (Figure 2a and 2b). Under oral challenge, infection-free samples raised from 6/48 to 33/48 in control populations, and from 11/48 to 35/48 in selected populations. As for systemic infection, samples without bacterial counts increased from 0/48 to 11/22 in control populations, and 0/48 to 22/48 for selected populations.

### 3. Correlated responses to selection of alternative routes of infection

We wondered how much of the adaptation to one route of infection would protect individuals infected through a different route. To address this, individuals of both sexes from control and selected populations were infected by pathogens via each of the two alternative routes of infection at two different time points (generations 14–15 and 24–25).

For both the oral and systemic infection treatments, there was a significant overall interaction effect between sex, selection regime and generation (χ^2^_6_ = 67.795 and χ^2^_6_ = 15.420, *P*<0.0001 and *P*<0.05 for oral and systemic infections, respectively). We therefore compared the hazard ratios between the selection regime and their respective controls, independently for the two time points and averaging the effect of sex.

**Figure 3:**
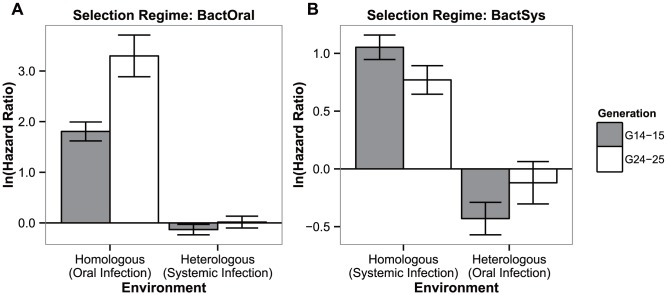
Test of adaptation and its correlated response. Hazard ratios of lines evolving in presence of a pathogen relative to controls at generations 14–15 (grey bars) and 24–25 (white bars) of adaptation, when exposed to the challenge they have evolved with or to the other infection route. (a) Oral infection selection regime (BactOral) and (b) systemic infection evolved flies (BactSyst). All populations spent one generation in a common environment before being tested. Vertical bars correspond to the standard error of the estimated ratio between the two selection regimes.

Concurrently with the survival data obtained for generations 14–15 and 24–25 in Figures 1a and 1b, evolved populations tested in the conditions in which they evolved (hereafter homologous environment) had a significantly higher survival relative to their controls. This is shown by the significant departure from zero of their hazard ratios (Figure 3: oral infection: |z|>8.003, *P*<0.001 in both generations; systemic infection: |z|>6.229; *P*<0.0001 in both generations). In contrast, exposing the adapted populations to the challenge they have not evolved in (hereafter heterologous environment), revealed no difference between control and selected lines for the BactOral selection regime (|z|<1.292, *P*>0.784 in both generations). For the BactSys selection regime, a significant difference was found in generations 14–15 (in which Bactsys<control), but not in the later generations (|z| = 3.062, *P*<0.01, and |z| = 0.656, *P* = 0.939, respectively). Therefore, adaptation to *P. entomophila* through one infection route infection did not affect susceptibility to the same pathogen infecting from a different route.

### 4. Correlated responses to other pathogens

**Figure 4:**
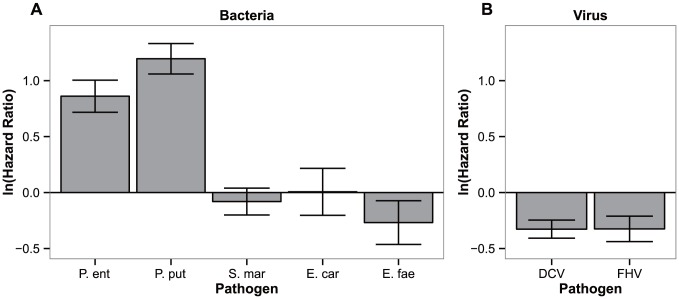
Specificity of the response. Differences in hazard ratios between control lines (ContSys) and evolved lines with *Pseudomonas entomophila* systemic infection (BactSys), when exposed to (a) bacterial pathogens, P.e (*P. entomophila*), P. put (*Pseudomonas putida*), S.mar (*Serratia marcescens*), E.fae (*Enterococcus faecalis*); and (b) viral pathogens, DCV (Drosophila C Virus), FHV (Flock House Virus). Vertical bars correspond to the standard error of the estimated ratio between the selection regime and controls.

Subsequently, we tested whether specificity of the evolved response could extend to other pathogens when infected via the same route (Figure 4).

Hazard ratios between the BactSys and ContSys populations after infection with the closely related species (same genus) *P. putida* were equivalent to those obtained with the original challenge, *P. entomophila* (|z| = 6.001 and 8.790, for *P. entomophila* and *P. putida*, respectively, *P*<0.001 in both comparisons). In contrast, challenges with other known *Drosophila* pathogens such as *Serratia marcescens* and *Erwinia carotovora*, also Gram-negative Gammaproteobacteria, or *Enteroccocus faecalis*, a Gram-positive bacterium, caused equal degrees of mortality between evolved populations and their controls (|z| = 0.670, *P* = 0.503; |z| = 0.031, *P* = 0.976 and |z| = 1.374, *P* = 0.170 for *S. marcescens*, *E. carotovora and E. faecalis*, respectively). We therefore conclude that the response obtained is specific, at least, to the *Pseudomonas* genus level but not for all Gammaproteobacteria. Finally, fly populations evolving with *P. entomophila* infection were more susceptible than control populations to infections with Drosophila C Virus (DCV) and Flock House Virus (FHV) (|z| = 4.043 and 2.855, *P*<0.001 and *P*<0.05 for DCV and FHV infections respectively).

## Discussion

Here, we report the first study addressing the impact of different infection routes taken by horizontally-transmitted pathogens on the evolutionary trajectories and outcomes of their hosts. Our main conclusions are:

- both exposure to systemic or oral infection results in the evolution of resistance in hosts, albeit at a different pace;
- adaptation is route-specific: hosts that adapt to pathogens from one infection route do not become less susceptible to the same pathogen infecting through a different route;
- the populations that evolved under systemic challenge by *P. entomophila* do not exhibit a generalized response outside the *Pseudomonas* genus; rather, resistance to this bacteria trades off with survival to infection with viruses.

### Different genetic bases for adaptation to distinct infection routes

Despite using the same pathogen in both infection protocols, we observed a lack of cross-resistance after a heterologous challenge with the same pathogen. Indeed, fly populations adapted to an oral infection by *P. entomophila* are equally susceptible to a systemic infection by the same bacterium species as populations evolved without the pathogen. The same holds true for populations evolved under a systemic infection challenged with an oral infection. This indicates that the response to each challenge has a different genetic basis.

Several genes and pathways are known to specifically participate in each infection route [23], [25], [33], [40] and our results are compatible with these findings. Yet, both humoral and epithelial responses may lead to the activation of anti-microbial peptides (AMPs) [25], [36], [49]. Moreover, the same pathways may be activated and required for survival in both infection routes. For instance, the Imd pathway has a role in protection against both orally and systemic infection with *P. entomophila* [38], [50]. Therefore, some of these effector elements could constitute a common target for selection and a general basis for adaptation to the pathogens, irrespective of infection route [51]. This is probably not the case, otherwise we would observe a positive correlation among responses.

### A rapid response

A few studies have previously shown that evolution of the response to different pathogens in *Drosophila* occurs at a rapid pace [44], [46]. Our results confirm this rapid evolution but they also show that the rate of adaptation is contingent upon the infection route taken by this pathogen. Specifically, the increase in survival to oral infection in our fly population occurs within fewer generations than the response to systemic infection, and it reaches a higher plateau. Because this is the first study that compares adaptation to different infection routes, whether these differences in dynamics are a general feature remains to be established. It would be interesting in the future to compare other pathogens that can infect through these different routes.

The observed differences in the evolutionary dynamics of populations exposed to each challenge may be due to the different genetic bases underlying each adaptation process. However, other factors may account for different dynamics. For example, systemic infection may be associated with more environmental variance (Ve) than oral infection. These differences in Ve would lead to the observed differences in dynamics even in the absence of different genetic bases for the traits underlying adaptation to each challenge. Quantitative genetic designs allowing measures of environmental and additive genetic variance for these traits are needed to distinguish between such alternatives.

### Evolution of resistance

Interestingly, in our experiments the only aspect in which the adaptive responses to oral or systemic infections are parallel, regards the evolution of resistance (Figure 4a and 4b). Indeed, we find a significant difference between the bacterial counts of control and evolved lines at the onset of mortality for each selection regime. At a later time point (120 h), control and evolved flies have the same bacterial load. However, at this point, we are only measuring bacterial loads in flies that survive infection, hence this information is irrelevant to the clarification of the mechanism involved in the adaptation process. Our results thus reiterate the need to follow the infection dynamics to discriminate between resistance and tolerance. Yet, with our data, we cannot exclude a role for tolerance, as the infected flies from evolved and control populations that survive may have different abilities to cope with the infection (e.g., in terms of fecundity or subsequent mortality). Given that theory predicts different evolutionary outcomes depending on whether host responses involve tolerance or resistance [52], it is important to establish experimentally which of these mechanisms is acting in an evolving population.

The similarity observed among responses to each challenge does not imply an equivalence of mechanisms. The clearance of bacteria in fed versus pricked flies is likely bound to rely upon very different processes [33]. Bacterial loads are much lower in orally infected flies (two orders of magnitude) than in systemic infections (compare panels a and b of Figure 4), despite the fact that in the oral infection treatment the bacteria density administrated was four orders of magnitude higher than in systemic infections, indicating that elimination mechanisms are much more effective in this route of infection. This is consistent with published work showing that oral infection provokes strong epithelial responses namely by the modulation of physical barriers blocking pathogen access to the body cavity and of gut epithelium renewal, and there is limited crossing of the bacteria to the body cavity [33], [40], [41], [53]. In contrast, in a systemic infection the pathogen is inside the body cavity. Thus, any reduction in pathogen loads in the populations adapted to systemic infection must rely on active methods of identifying and eliminating bacterial invaders, namely through the canonical action of AMPs and plasmatocytes [23], [25], [42].

### Pathogen specificities

The evolved populations only respond to infections with the bacterium used for selection, *P. entomophila*, and to its close relative *P. putida*. Other bacteria cause the same levels of lethality as in controls. This genus-specific response is somewhat surprising in that systemic infection with different bacteria can induce a wide-spectrum of AMPs and other immune responsive genes with large overlaps, yet closely related pathogens induce considerably divergent responses [54], [55], [56]. Other studies using inbred lines have also established a lack of correlation between bacterial loads of different bacteria [57]. Finally, this specific adaptation to the *Pseudomonas* genus comes at a cost in survival to viral infections (Figure 3). Other studies provide contradictory evidence regarding the existence of trade-offs between susceptibility to different pathogens [54], [58], [59], [60]. This study, however, strongly points to the occurrence of a trade-off, where adapting to one pathogen increases susceptibility to others. This trade-off may underlie the maintenance of variation for resistance to *Pseudomonas* in the population.

### Implications for the evolution of host-pathogen interactions

Several studies have shown that infection routes affect the evolution of virulence in pathogens [4], [5], [6], [7], [11]. Here, we show that host adaptation to pathogens is also contingent upon those infection routes. Therefore, host responses may confound the conclusions drawn from studies on the evolution of virulence in pathogens in natural populations. For example, most pathogens that infect invertebrate hosts systemically are transmitted by vectors [14]. Several factors are expected to differentially affect virulence in vector-borne or directly-transmitted pathogens [8], [9], [10]. However, here we show that hosts adapt slower to a systemic than to an oral infection. This may confound the conclusions drawn from the observation of virulence patterns in natural populations. Hence, instead of merely observing patterns, studies on the effect of transmission modes in the evolution of host-pathogen interactions should follow the processes of adaptation in hosts and pathogens separately, to pinpoint the real cause underlying the observed patterns. In this sense, experimental evolution is a powerful, yet underexploited tool to unravel the selection pressures underlying host-pathogen interactions. Our findings reinforce the necessity of including the mechanism of pathogen access into the set of criteria used to categorize and study host-pathogen interactions in ecological immunity, physiology and evolution [14], [16].

## Materials and Methods

### Foundation and maintenance of *Drosophila melanogaster* populations

An outbred population of *Drosophila melanogaster* was established in the laboratory in 2007, from 160 *Wolbachia*-infected fertilized females, collected in Azeitão, Portugal. Variability in this base population was assessed using multiple methods, based on 103 SNPs located in the left arm of the 3rd chromosome (supplementary methods). It contains high and relatively constant levels of polymorphism (SI, Figure S1). The population was kept in the laboratory cages for over 50 non-overlapping generations (generation time: three weeks) with high census (>1500 individuals). Flies were maintained under constant temperature (25°C), humidity (60–70%) and light-darkness cycle (12∶12), and fed with standard cornmeal-agar medium. Prior to the initiation of experimental evolution, the initial population was serially expanded for 2 generations to allow the establishment of 16 new populations used in this work (see below).

### Pathogen cultures

Experimental evolution of *D. melanogaster* populations was performed using *Pseudomonas entomophila*. In addition, we used other pathogens in some assays, namely, *Pseudomonas putida, Serratia marcescens, Erwinia carotovora, Enterococcus faecalis*, DCV (Drosophila C Virus) and FHV (Flock House Virus). For each round of infections, bacterial pathogens were grown in LB inoculated with a single bacterial colony, taken from solid medium cultures grown from glycerol stocks kept at −80°C and streaked in fresh (<1 week) Petri dishes. Excluding *P. entomophila*, grown at 30°C, all bacteria were prepared from an overnight culture grown exponentially at 37°C, centrifuged and adjusted to the desired OD (see below). The *P. entomophila* strain used for experimental evolution was a generous gift from Bruno Lemaitre. It is resistant to rifampicin, which was used as a marker trait. The remainder bacterial pathogens were generous gifts from Karina Xavier (*P. putida*), Dominique Ferrandon (*S. marcescens*) and Thomas Rival (*E. carotovora* and *E. faecalis*). Viruses were produced as described elsewhere [61] and aliquots were kept at −80°C and thawed prior to infection.

### Experimental evolution

Lines of all treatments were derived from the same base population (four lines per treatment). Four selection regimes were created, to which the following treatments were applied: systemic infection, in which flies were pricked in the thoracic region [32] with *P. entomophila* (OD600 = 0.01) (BactSys regime); a control for injection, following the same procedure except that the needle was dipped in sterile LB as a control (ContSys regime); oral infection, in which the food plates were covered for 24 hours with filter papers soaked with a *P. entomophila* culture (OD600 = 100) diluted 1∶1 with sterile 5% sucrose solution (BactOral regime) (adapted from [41]); and control lines, where flies were kept in standard food (Control regime). The dose of *P. entomophila* for both bacterial treatments was determined at the start of the selection experiment to cause an average mortality of 66% in the base population, which corresponds to an OD of 0.01 for the systemic and of 50 for the oral infection treatments, respectively (SI, Figure S2).

These treatments were administrated at each generation to 310 males and 310 females (4–6 days old since eclosion). The subsequent generation was founded by all survivors at days 5 and 6 after treatment. The density of eggs was limited to 400 eggs in each cup, a density determined experimentally to enable optimal larval development. Each generation cycle lasted 3 weeks. Absence of transmission of the pathogen to the progeny was tested by plating whole pupae homogenates in LB agar plates supplemented with 100 µg/ml rifampicin. No evidence of transmission of the pathogen to the next generation was found for either infection route, as plating of the progeny of infected flies (pupae) resulted in no *P. entomophila* colony. Altogether, populations evolved in their specific treatments for 24 generations in the case of the BactOral regime and 34 generations in the case of the BactSys regime.

At each generation, a sample of individuals from each selection regime was used to monitor survival across generations. To this aim, individuals from each replicate population of the BactSys and the ContSys selection regimes were exposed to systemic infection with *P. entomophila*, whereas individuals from the BactOral and ContOral selection regime were exposed to oral infection with the same bacteria species, and their mortality was monitored in vials for at least 10 days. For systemic infections, 100 individuals were placed in vials of 10 individuals. For the oral infection treatments, 120 individuals were placed for 24 hours in groups of 20 in vials where the food was covered with a filter paper disk soaked in bacteria solution, and subsequently transferred to standard food vials. A mixed sample of 200 individuals of the four populations of the Control selection regimes (ContSys and ContOral) were used as controls in these experiments. To further confirm that persistent infection was not affecting the results, e.g., due to immune priming, at generation 20, these tests were also performed using individuals whose eggs were previously decontaminated in 50% bleach for 2 minutes. Evolved populations showed the same proportion of individuals surviving after infection with/without bleaching.

### Pathogen loads in controlled and selected populations

*P. entomophila* quantifications were performed in two assays at generations 23 to 25, as described in Nehme et al (2007) [30] with minor modifications. For these assays, 150 to 250 flies (males and females) from each control and selected population were infected as in the survival assays. Flies were collected at 14 and 120 hours after systemic infection for BactSys and ContSys regimes, and at 40 and 120 hours after oral infection, for the BactOral and Control regimes. These time points were selected as the ones that correspond to the point before the onset of mortality in both modes of infection, and to the first day of egg-laying, after which no further mortality occurs (Figure S2). Six replicates of pools of 3 infected flies were homogenized in 50 µL of sterile 1 mM MgCl medium and serially diluted. Homogenates (4 µl) were plated in triplicate on LB agar plates, supplemented with 100 µg/ml Rifampicin and incubated overnight. The next day, we counted the number of colony-forming units (CFUs) on those plates. To avoid possible artifacts due to different maternal effects, flies used in these tests were the progeny of unselected flies that spent one generation in a common environment.

### Adaptation and its consequences in heterologous environments

To test how host adaptation to pathogens from one infection route affected the host response to pathogens from a different route, 100 individuals (males and females) from each of the replicate populations of the BactSys and BactOral selection regimes, and the matching controls were exposed to the environment they evolved in as well as to that of the heterologous selection regime (orthogonal assay), following the same protocol of the survival assays, at generations 15 and 25. To avoid possible artifacts due to different maternal effects, flies used in these tests were the progeny of flies that spent one generation without being exposed to pathogens, thus all in the standard environment of the base population.

### Testing the generality of the response

To test how adaptation to a specific pathogen affected host responses to other pathogens, 100 individuals (males and females) from each replicate population of the BactSys and ContSys selection regimes were systemically infected with the following pathogens: *Pseudomonas putida* (OD_600_ = 10); *Serratia marcescens* (OD_600_ = 0.01); *Erwinia carotovora* (OD_600_ = 150); *Enterococcus faecalis* (OD_600_ = 3); DCV (TCID_50_ = 2×10^7^); FHV (TCID_50_ = 5×10^6^). These tests were performed between generations 27 and 30, and were repeated at least twice for each pathogen. The protocol followed was the same as that used for the cross-testing experiments. We could not perform this experiment with oral infections because we were unable to find another pathogen that caused mortality in our population via this infection route.

### Statistics

All statistical analyses were done using R (v 2.15).

To compare survival across generations in flies evolving with or without pathogens, the proportion of individuals surviving at day 10 after infection in each vial was first estimated using the Kaplan-Meier method. Individuals alive at the end of the experiment, stuck in the food or escaped from vials during the period of observation were counted as censored observations. Afterwards, the square root of the proportion of surviving individuals was arcsin transformed and analyzed using a general linear mixed model, with sex, generation and selection regime as fixed factors and replicate population as a random factor. To test for the effect of the selection regime, a model with sex and generation as fixed factors was compared with a model with sex, generation and selection line as fixed factors. To test the different effects of the selection line across generations a model with interaction between selection line and generation was compared with the model without this interaction. To compare the proportion of individuals surviving at each generation, each selection regime was contrasted with its control at a given generation and corrected for multiple comparisons using the Bonferroni correction.

To compare survival between the control and selected population in the homologous and in heterologous selection environment, and after infection with different pathogens, we used a Cox's proportional hazards mixed effect model. The model included sex, selection regime and generation as fixed factors and test vials nested into population as random factor, thus accounting for variation in survival rates between populations within each selection line and between vials [62].

To compare pathogen loads, a linear mixed model on the natural logarithm of bacterial counts was employed, with selection regime, time after infection and sex as fixed factors and population as random factor. Interactions among all fixed factors were included in the full model, and sequentially removed if non-significant (*P*>0.05). These tests were done using the R libraries *lme4* (v0.999999, generalized and linear mixed models), *coxme* (v2.2, mixed effects Cox proportional hazards model) and *glht* (v1.2, multiple comparisons).
